# Intervention Effects on Physical Activity and Insulin Levels in Men of Pakistani Origin Living in Oslo: A Randomised Controlled Trial

**DOI:** 10.1007/s10903-012-9686-3

**Published:** 2012-07-25

**Authors:** Eivind Andersen, Arne T. Høstmark, Ingar Holme, Sigmund A. Anderssen

**Affiliations:** Department of Sport Medicine, Norwegian School of Sport Sciences, Ullevaal Stadium, Box 4014, 0806 Oslo, Norway

**Keywords:** Minority group, Glucose, Insulin, Physical activity

## Abstract

High prevalence of type 2 diabetes (T2D) is seen in some immigrant groups in Western countries, particularly in those from the Indian subcontinent. Our aims were to increase the physical activity (PA) level in a group of Pakistani immigrant men, and to see whether any increase was associated with reduced serum glucose and insulin concentrations. The intervention was developed in collaboration with the Pakistani community. It used a social cognitive theory framework and consisted of structured supervised group exercises, group lectures, individual counselling and telephone follow-up. One- hundred and fifty physically inactive Pakistani immigrant men living in Oslo, Norway, were randomised to either a control group or an intervention group. The 5-month intervention focused on increasing levels of PA, which were assessed by use of accelerometer (Actigraph MTI 7164) recordings. Risk of diabetes was assessed by serum glucose and insulin concentrations determined in a fasted state, and after an oral glucose tolerance test (OGTT). ANCOVA was used to assess differences between groups. There was a mean difference in PA between the two groups of 49 counts per minute per day, representing a 15 % (95 % CI = 8.7–21.2; *P* = 0.01) higher increase in total PA level in the intervention group than in the control group. Insulin values taken 2 h after an OGTT were reduced in the intervention group by 27 % (95 % CI = 18.9–35.0; *P* = 0.02) more than those in the control group. There were no differences in fasting or postprandial glucose values between the groups at the follow-up test. This type of intervention can increase PA and reduce serum insulin in Pakistani immigrant men, thereby presumably reducing their risk of T2D.

## Background

Immigrants comprise an increasing proportion of many Western populations. In Norway, the immigrant population is predicted to increase from currently 11 % of the population, to 22–28 % of the population in 2060 [1]. A higher prevalence of type 2 diabetes (T2D), which is also a main risk factor for cardiovascular disease (CVD), is seen in some immigrant groups. It seems that those who have migrated from the Indian subcontinent (Pakistan, India and Bangladesh) to Western countries, and their descendants, are particularly vulnerable to this disease [2–5]. The reason for this apparent higher disease risk is not known, but is probably attributed in part to an excess of insulin resistance [4, 6, 7]. Physical inactivity seems more prevalent among South Asian (SA) immigrants than in the native population [8–11] and is, together with central obesity [12], highly likely to contribute to insulin resistance [13]. A higher prevalence of T2D and other CVD risk factors among immigrants living in their new country compared with those from their country of origin may support the hypothesis that a change in lifestyle, such as lowered physical activity, is a causative factor [2, 14]. While physical inactivity may be an independent risk factor for T2D, several studies suggest that physical activity (PA) may protect against developing T2D [15–17].

Little work has been done to understand why so many immigrants from SA countries are physically inactive. Lack of knowledge of the health effects of PA, socio-economic factors, language barriers and religious beliefs are some of the factors that have been linked to the PA behaviour in this group [18, 19]. The few interventions that have targeted ethnic minority populations, with most of these demonstrating disappointing results, have had poor methodological quality (e.g. a non-randomised design or small sample size) [18, 20]. However, a common feature of the interventions that showed improvements in PA with immigrants was the input of community participants’ expertise in both defining strategies and shaping interventions [18, 20].

Social cognitive theory (SCT) has been acknowledged as one of the leading health behaviour change theories used to explain and predict changes in PA [21, 22]. Key SCT constructs applied to PA include the physical environment (i.e. opportunities to do PA), the social environment (i.e. social support for PA, and physically active role models), self-efficacy (i.e. confidence to do PA), outcome expectations (i.e. the values associated with being physically active), behavioural capability (i.e. knowledge and skill to do PA) and self-control (i.e. personal goal setting and monitoring of PA). However, little work has been done to apply these constructs to the PA of immigrants from SA countries. SCT-based strategies to change behaviour can include providing opportunities and social support, promoting mastery learning through skills training and approaching behaviour change in small steps, providing opportunities for goal setting and training in problem solving, and correcting misperceptions and presenting outcomes that have functional meaning. Although there is promising evidence to support the use of SCT in PA interventions, most of the behaviour change literature is based on Caucasians and may not be applicable to ethnic minorities or immigrants [23].

Because we have little knowledge of the PA behaviour in this group of men and ethnic minorities in general, a participatory approach in the development of the intervention was considered necessary. The aims of this study therefore were to target Pakistani immigrant men living in Oslo and (1) to use a participatory approach to identify SCT influences and strategies to develop an intervention to increase PA, (2) to examine the efficacy of the intervention in increasing PA using a randomised controlled design, and (3) to examine whether increasing PA would be associated with reduced plasma glucose and insulin concentrations, thereby potentially lowering the risk of T2D.

## Methods

### Formative Research: Physical Activity Influences

To better understand why many Pakistani immigrants are physically inactive and how to positively influence their PA behaviour, we conducted two focus groups with representatives from the male Pakistani immigrant group (*n* = 10 in each group, age ranged from 25 to 60 years). Each focus group meeting lasted approximately 2 h. The aims of these group meetings were to explore expectations, expectancies, preferences and barriers to PA among the Pakistani immigrant men. These discussions revealed that: the men had very few physically active friends or family members, had little knowledge about non-vigorous PA and the link between PA and health and staying regularly physically active, had many barriers to PA (e.g. managing time) and did not know if they were able to overcome them, and they did not see many benefits of being regularly physically active. Based on these results, we decided to target the following SCT key concepts to promote PA change: environment, behavioural capability, self-control, self-efficacy, expectations and expectancies.

### Participants

**Fig. 1:**
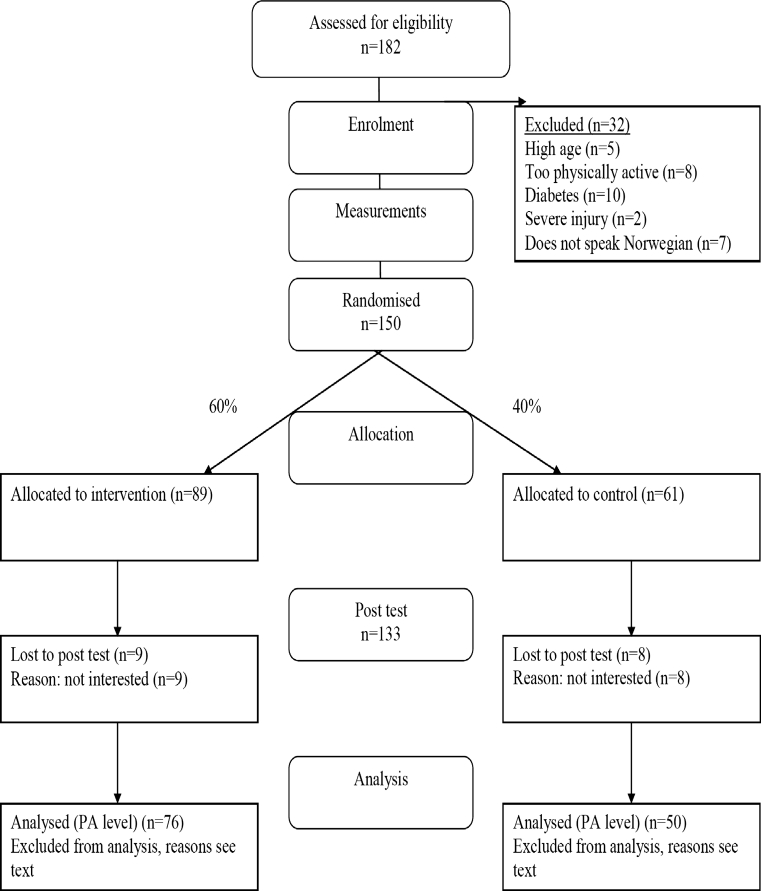
The flow of participants through the trial

Men living in Oslo with a Pakistani background (either born in Pakistan or having had both parents born in Pakistan) in the 25–60 year age group, who were not physically active on a regular basis (exercising at most twice per week at a moderate or higher intensity level for 30 min or more at a time, or were active commuters) were candidates for inclusion in this study. Those excluded from the study were subjects with known diabetes, with injuries that would make it difficult to participate in organised exercise sessions, or who did not to speak Norwegian. The recruitment process was carried out during the autumn of 2008. We gave a brief oral presentation concerning the project at six mosques and at various Muslim festivals in Oslo. Before participation in the study, written informed consent was obtained from each participant (see Fig. 1 for participant flow through the study). The Regional Committee for Medical Research Ethics (Ref. no. S-07300b) and the Norwegian Social Science Data Services (Ref. no. 17212/2/KS) approved the study.

### Intervention Programme

In close collaboration (five representatives of the group were members of the steering committee) with representatives from the target group, we developed an intervention to target key SCT constructs via the following components: structured group exercise sessions twice a week led by an exercise physiologist, two group lectures, one individual counselling session, written material and a phone call. Table 1 provides an overview of how these components were conceptualised with reference to SCT. The intervention programme lasted 5 months. The control group members received their baseline results approximately 2 weeks after the testing, and were offered organised exercise, one group lecture and written material following completion of the intervention period.

**Table 1:** Overview of the intervention components, the behaviour change strategies and the targeted constructs

Intervention component	Dose	Description	Behaviour change strategy	Targeted construct
Structured group exercise	60 min 2/week	Participants could choose to attend one of five different exercise facilities in Oslo. The different exercise groups were led by an exercise physiologist. The exercise training programme was designed as a low-threshold activity. The sessions had the following structure: a 15 min warm-up with easy and fun games, 40 min of floor ball and/or football plus some strength exercises and a 5 min cool down. Seven participants did not attend any of the sessions (one trained by himself and six were not motivated) and two were injured at the first exercise session. The mean attendance was 60 % (range: 11–100 %)	Provide opportunities for PA Increase social support for PA Promote mastery learning through skill training Improve knowledge and skill to perform PA Promote positive outcomes Provide credible role models Social modelling	Environment Behavioural capability Expectancies Self-control Self-efficacy
Group lectures	2 × 2 h	Major topics: What is PA? PA and health link; short- and long-term effects The harms of physical inactivity PA recommendations and how to achieve these Activity examples Setting small goals Identifying and reducing perceived barriers Making a PA plan Seeking social support Self-reward Both attendees and non-attendees received written summaries of the lecturers	Improve knowledge of PA options, including non-vigorous PA Improve knowledge of how to incorporate PA into a daily routine Enhance PA expectancies Improve goal setting Improve problem solving of PA barriers Improve social support for PA	Behavioural capability Expectancies Self-control Self-efficacy
Individual counselling sessions	1 × 1 h	All participants completed this part of the intervention. The counselling was based on the concept that all advice must match the participants’ experiences of PA and their degree of motivation. Together with the participant, the primary goal was to find activities that could be implemented in a normal week, with the sum of these activities enabling them to reach the PA recommendations. After discussing activity options, the participants set the goals they wanted to achieve over the 5-month period. Finally, we discussed barriers by asking “What do you think can stop you from carrying out this activity plan?”, and the possible barriers, and solutions to them were discussed and written down	Identify opportunities for PA Improve knowledge and skill to perform PA Enhance goal setting Enhance problem solving Promote mastery Identify and problem solve barriers to PA	Environment Behavioural capability Self-control Self-efficacy
Phone call	1 × 5–15 min	3–5 weeks before the post-test, intervention participants were telephoned. The focus of this conversation was to discuss the activity plan, to make changes if necessary, and to encourage further efforts. All participants were reached within three attempts	Provide feedback on PA behaviour Reinforce problem solving Provide encouragement and help	Social support Self-control Self-efficacy

### Experimental Design

Participants (*n* = 150) were randomly assigned to either the intervention group or control group using a random computerised list. The randomisation ratio was 60:40, meaning that each participant had a 60 % chance of being allocated to the intervention group. We chose this randomisation ratio in anticipation of a high dropout rate in the intervention group. We also wanted a higher power to show internal relations within the intervention group. The participants were randomly allocated to one of the two groups on the same day as the baseline assessments. Neither the test personnel nor the participants knew which group they had been assigned to until the baseline testing was finished. The staff members involved in the intervention had to be aware of the group assignments; thus, the study was not blinded. However, the laboratory staff and data-entry personnel did not know the participants` group assignments.

### Adverse Effects

Throughout the intervention period there were two serious injuries (a broken leg and a disk herniation) in addition to some minor back problems. The injuries are not believed to have been caused by the exercise programme per se.

### Measurements

Each participant was examined for PA habits and diabetes risk factors both before and immediately after the 5-month intervention. All testing was conducted at the Norwegian School of Sport Sciences. The baseline test was carried out in October–November 2008 and the post test in March–May 2009. After a 12-h overnight fast (minimum 8 h), venous blood samples were drawn from an antecubital vein. An oral glucose tolerance test was performed, i.e. 75 g glucose in 200 mL of water was ingested and plasma glucose, insulin and C-peptide were determined before and 2-h after ingestion of the glucose drink. The samples were centrifuged for 10 min at 2,500*g* (except for HbA1c) and analysed the same day.

Blood variables were determined at the Dr. V. Furst Laboratory for clinical chemistry, Oslo, Norway. A Modular P Machine (Roche, Japan) was used for measuring glucose (photometry), insulin and C-peptide (immunoassays). Glycosylated haemoglobin (HbA1c) was measured on an HPLC, G7 instrument (Tosah, Japan).

The degree of insulin resistance was estimated by HOMA (homeostasis model assessment) according to the method described by Matthews et al. [23]. Insulin resistance score (HOMA-IR) was computed with the formula: (*fasting plasma glucose x fasting serum insulin*)/22.5. Low HOMA-IR values indicate high insulin sensitivity, whereas high HOMA-IR values indicate low insulin sensitivity (insulin resistance).

Habitual PA was assessed with an MTI Actigraph accelerometer (MTI model 7164; Manufacturing Technology Inc., Fort Walton Beach, FL, USA). This is a seismic instrument that continuously measures acceleration, raw data from this instrument are called “counts”, which are the sum of acceleration in a given time period. The participants were instructed to wear the accelerometer for 7 days on the right hip during all waking hours, except while swimming and bathing. Accelerometers were programmed to start recording at 6 am the day after the participants received their accelerometer. The epoch length was set to 1 min. In the analysis of accelerometer data, epoch periods with a value of zero (with two exceptions) for 60 min or longer were interpreted as “accelerometer not worn” and excluded from the analysis [24, 25]. Physical activity data were included if the participant had accumulated a minimum of 480 min of activity data per day for at least 2 days, regardless of type of day (work day or week day). There were no differences in counts per minute per day (CPM) between those who wore the monitor for 2 days and those who wore the monitor for 3 days or more. For that reason, it was decided to also include those participants who had only worn the monitor for 2 days (baseline; *n* = 7, post-test; *n* = 3). On average (±SD) participants wore the monitor for 6.3 ± 1.8 days at baseline and 6.1 ± 1.5 days at the post-test assessment. Average wearing time (±SD) was 13.5 ± 1.5 h day^−1^ at baseline and 13.6 ± 1.6 h day^−1^ at the post-test assessment. There were no differences between the two groups in wearing time. Accelerometer data were processed and analysed using the SAS-based (version 9) software program (SAS Institute Inc. Cary, North Carolina, USA) called CSA-analyser (http://csa.svenssonsport.dk). One hundred and forty-two participants had valid recordings at the baseline test (95 %). Four lost their monitor and four had less than two valid days of recordings. At the post-test, 126 participants (84 %) had valid recordings (intervention group *n* = 76, control group *n* = 50), 17 were lost to the post-test, five had less than 2 days of recordings, and two did not return their accelerometer. The primary outcome variable from the accelerometers is average CPM throughout the measurement period. Secondary outcomes are the minutes spent in various levels of PA intensity in which sedentary behaviour (inactive time) was defined as ≤100 counts/min [24], light intensity activity as 101–1,951 moderate intensity as 1,952–5,724, vigorous intensity as 5,725–9,497 and any amount above 9,497 is considered very vigorous intensity [26]. These cut points are widely used and show a good correlation (0.88) with direct VO_2_ measurement [26].

Aerobic fitness or Peak VO_2_, defined as the highest measured VO_2_ (mL kg^−1^ min^−1^), was assessed through a maximum exercise test on a treadmill. We used a modified Balke protocol [27]. Gas exchanges were continuously sampled in a mixing chamber every 30 s by the participant breathing into a Hans Rudolph two-way breathing valve (2,700 series, Hans Rudolph Inc., Kansas City, USA). The breathing valve was connected to a Jaeger Oxycon Pro (Erich Jaeger GmbH, Hoechberg, Germany) and used to analyse the oxygen and carbon dioxide content. The analyser was volume- and gas-calibrated before each test. The test result was approved when scoring ≥16 on the Borg 6–20-point rating of perceived exertion scale and when the respiratory quotient was >1.1. For safety reasons, we tested only those younger than 40 years (*n* = 99).

Waist circumference was measured at the end of a gentle expiration, in a straight line to the chest, midway between the lower rib margin and the iliac crest. Weight was measured without shoes in light clothing by a SECA electronic scale (SECA model 767, Germany) to the nearest 0.5 kg. Height was measured without shoes with a transportable stadiometer (Harpenden; Holtain, Crymych, GB) and set to the nearest 0.5 cm.

### Sample Size Calculation

In the sample size calculation, we used PA (CPM) as the primary outcome variable. A difference in group means of 60 CPM was considered an important change (approximately 10–15 %). Based on data from a PA intervention conducted on obese children (data not published), a standard deviation of 120 was chosen. With a power of 0.8, a significance level of 0.05 and a presumed drop-out rate of 20 %, a total of 144 participants were needed.

### Statistical Analysis

Means (±SD) were used to describe baseline data. Independent samples *t* test was used for testing differences between groups at baseline. The response to the intervention was measured as the difference between the corresponding final and baseline values for all variables (post-baseline; per protocol analysis without imputations). Repeated measures ANCOVA was used for analysing mean changes within each group and for testing differences between mean changes in the two groups. All analyses were adjusted for age and baseline differences. Effect sizes (ES) were calculated as: (changes in the control group—changes in the intervention group)/SD in the control group. The associations between each exposure (i.e. changes in total PA and inactive time) and changes in the outcome (insulin-2 h) were examined in univariate analyses using linear regression analyses. Multivariate regression analyses were used to adjust for waist circumference when looking at the relationship among changes in total PA, inactive time and changes in insulin-2 h. We analysed all data using the Statistical Package for the Social Sciences (SPSS, IBM Inc. Chicago, USA) version 15.

## Results

Seventeen (11 %) participants were lost to the post-test. Nine were lost from the intervention group and eight from the control group. The main reason given for not attending the post-test was lack of interest. There were no differences between drop-outs in the two groups on any variable. Except for a lower baseline PA level (CPM), the drop-outs were no different on any variable than those who completed both the baseline and the post-test.

Table 2 displays the baseline characteristics of both the control and intervention groups. A mean difference in age and glucose-2 h levels between the two groups was observed. Furthermore, a difference of 46 CPM was observed in the total PA level, which translates into a 15 % (95 % CI = 9.1–20.8; *P* = 0.05) higher PA level in the intervention group. However, there was no difference in total PA level (CPM) between the groups when adjusted for age (mean difference = 40, 95 % CI = −5.6–85; *P* = 0.08). The amount of moderate to vigorous PA (MVPA) was reasonably good, but of the 142 participants with complete accelerometer data, only six (4.2 %) reached the PA recommendations of 30 min of MVPA per day (PA sessions had to be of at least 10 min duration or more). Average fasting and postprandial glucose levels were within the normal range in both groups. Ninety-six per cent had insulin-2 h levels above the health related range (18–173 pmol/L), and 93 and 81 % were classified as overweight according to BMI ≥23 [38] and waist circumference ≥90 cm, respectively. Eighty three per cent of the men were not born in Norway.

**Table 2:** Baseline values of the primary and secondary variables in each group

Characteristic	Intervention group (*n* = 89) mean (SD)	Control group (*n* = 61) mean (SD)	Mean difference (95 % CI)
Age (years)	35.7 (6.1)	39.7 (9.2)	−3.9 (−6.6 to −1.2)‡
Weight (kg)	83.7 (12)	84.1 (14.4)	−0.3 (−4.7–4.1)
Height (cm)	174 (6.2)	174 (6.2)	0.6 (−1.3–2.7)
BMI (kg m^−2^)	27.1 (3.2)	27.4 (4.2)	−0.2 (−1.5–0.9)
Waist circumference (cm)	98 (9)	99 (11)	−1.1 (−4.6–2.3)
Total PA (CPM)^a^	328 (138)	281 (118)	46 (3–89)
Inactive time (h day^−1^)^a^	8.4 (1.6)	8.9 (1.5)	−0.5 (−1.03–0.04)
MVPA (min day^−1^)^a^	35 (21)	28 (19)	6.4 (−0.4–13)
Peak VO_2_ (mL kg^−1^ min^−1^)^b^	33.9 (5.2)	34.7 (6.5)	−0.7 (−3.4–1.9)
HbA1c (%)	5.6 (0.60)	5.7 (0.67)	−0.1 (−0.3–0.1)
Glucose (mmol/L)	5.3 (0.7)	5.5 (1.1)	−0.1 (−0.5–0.1
Glucose-2 h (mmol/L)	6.4 (2.2)	7.6 (3.7)	−1.2 (−2.3 to −0.1)
Insulin (pmol/L)	101 (53)	107 (61)	−6 (−25–13)
Insulin-2 h (pmol/L)	750 (607)	865 (553)	−114 (−305–76)
C-peptide (pmol/L)	993 (296)	1,017 (346)	−23 (−131–83)
C-peptide-2 h (pmol/L)	3,688 (1,348)	4,057 (1,369)	−368 (−820–83)
HOMA-IR	4.0 (2.3)	4.2 (2.5)	−0.2 (−0.5–1.0)

*SD* standard deviation, *BMI* body mass index, *PA* physical activity, *MVPA* moderate, vigorous and very vigorous intensity physical activity, *HOMA*-*IR* homeostasis model assessment—insulin resistance

^a^ *n* = 59 and 83 for the control and the intervention groups, respectively

^b^ *n* = 30 and 69 for the control and the intervention groups, respectively

^‡^ *P* value: 0.05

The intervention group increased their total PA level (CPM) significantly more than the control group (Table 3). A mean difference between the two groups of 49 CPM was obtained, which translates into a 15 % (95 % CI = 8.7–21.2; *P* = 0.01) higher increase in total PA level in the intervention group than in the control group. Moreover, the participants in the intervention group increased their time in MVPA by 6.4 min more per day, than the control group. Both groups decreased their inactive time, but there were no significant differences between the two groups in this regard.

**Table 3:** Changes between post and baseline measurements in the intervention and control groups

Characteristic	Intervention group (*n* range; 69–77) ∆ mean (SEM)	Control group (*n* range; 47–53) ∆ mean (SEM)	Adjusted mean diff ±95 % CI^a^	Effect size	*P* value^a^
Weight (kg)	−1.7 (0.2)	0.1 (0.3)	−1.9 (−2.7 to −1.0)	−0.9	<0.01
BMI (kg m^−2^)	−0.5 (0.1)	0.3 (0.1)	−0.8 (−1.1 to −0.5)	−1.00	<0.01
Waist circumference (cm)	−1.9 (0.4)	1.7 (0.4)	−3.4 (−4.7 to −2.0)	−1.06	<0.01
Total PA level (CPM)	65 (12)	19 (13)	49 (83–9)	0.52	0.02
Inactive time (min day^−1^)	−13 (11)	−14 (15)	11 (−28–50)	0.1	0.5
MVPA (min day^−1^)	13 (2)	4 (2)	6.4 (0.5–12)	0.44	0.04
Peak VO_2_ (mL kg^−1^ min^−1^)^b^	7.3 (0.4)	3.7 (0.8)	3.6 (1.8–5.4)	1.06	<0.01
HbA1c (%)	0.06 (0.02)	0.04 (0.03)	−0.003 (−0.1–0.1)	−0.02	0.9
Glucose (mmol/L)	−0.14 (0.05)	−0.06 (0.1)	−0.1 (−0.4–0.1)	−0.09	0.3
Glucose-2 h (mmol/L)	−0.6 (0.2)	−0.6 (0.3)	−0.2 (−0.9–0.3)	−0.1	0.4
Insulin (pmol/L)	−15 (6.4)	−12 (5.8)	−5.5 (−24–12)	−0.1	0.5
Insulin-2 h (pmol/L)	−257 (65)	−59 (55)	−196 (−385 to −7)	−0.51	0.04
C-peptide (pmol/L)	−75 (36)	9 (33)	−88 (−195–18)	−0.37	0.1
C-peptide-2 h (pmol/L)	−573 (143)	−64 (153)	−445 (−886 to −6)	−0.42	0.04
HOMA-IR	−0.7	−0.5	−0.1 (−0.6–0.9)	−0.12	0.7

*SEM* standard error of the mean, *CI* confidence interval, *BMI* body mass index, *PA* physical activity, *MVPA* moderate, vigorous and very vigorous intensity physical activity, *HOMA*-*IR* homeostasis model assessment—insulin resistance

^a^Adjusted for age and baseline differences

^b^ *n* = 16 and 55 for the control and the intervention groups, respectively

Both groups reduced their insulin-2 h levels (Table 3). In the intervention group, insulin-2 h values decreased by 27 % more (95 % CI = 18.9–35.0) than in the control group. Similar findings were found for C-peptide-2 h (Table 3), in which the reduction was 14 % (95 % CI = 7.7–20.2) more in the intervention group. In addition, we found significant differences between the two groups in respect to changes in weight, BMI, waist circumference and peak VO_2_ (Table 3). However no effect on HOMA-IR and blood glucose was found.

In univariate analyses, reductions in insulin-2 h level were strongly related to changes in both total PA and inactive time (Table 4). The B-coefficients indicated reductions in insulin of 1.5 (pmol/L) for every increase in count per minute and a reduction in insulin of 1.6 (pmol/L) for every minute per day reduction in inactive time. Only minor changes in the relationship between total PA level and inactive time and insulin-2 h were seen when adjusting for changes in waist circumference (Table 4). There was no evidence of any association between changes in insulin-2 h and changes in either waist circumference, minutes in MVPA or peak VO_2_ (Table 4).

**Table 4:** Relations between changes in insulin-2 h and changes in PA variables and waist circumference

Independent variables	β coefficient (±95 % CI)	*t* value	*R* ^2^	*P* value
Univariate analyses (*n* = 102)				
Change total PA (CPM)	−1.5 (−2.4 to −0.5)	−3.2	0.091	0.002
Change inactive time (min day^−1^)	1.6 (0.7–2.5)	3.6	0.11	<0.001
Change MVPA (min day^−1^)	−4.5 (−10–1.7)	−1.4	0.01	0.1
Change Peak VO_2_ (mL kg^−1^ min^−1^)	−10 (−40–18)	−0.7	0.009	0.4
Change waist circumference (cm)	16 (−5.4–38.0)	1.4	0.019	0.14
Multivariate analyses^a^ (*n* = 102)				
Change total PA (CPM)	−1.4 (−2.4 to −0.4)	−3.0	0.10	0.003
Change inactive time (min day^−1^)	1.6 (0.72–2.5)	3.7	0.13	<0.001

*CI* confidence interval, *MVPA* moderate and vigorous intensity physical activity

^a^Adjusted for changes in waist circumference (cm)

## Discussion

In this randomised controlled study, Pakistani immigrant men increased their total PA level and reduced their waist circumference, as well as obtaining an appreciable reduction in insulin concentration following glucose ingestion. Presumably, these changes would imply a reduced risk of T2D, and it is conceivable that an increase in the amount of PA governed the beneficial changes in serum insulin.

The strengths of this study include the randomised and controlled design and repeated measures of PA with the use of accelerometers. The intervention group had a significantly higher PA level at baseline. In theory, this could mean a lower intervention potential, thus making our intervention effects more conservative in comparison with a situation in which the two groups were similar at baseline. Because persons who respond to this type of study may be motivated to increase their PA level, the external validity regarding wider populations may be questionable. Internally, however, a randomised design should prevent this from affecting the results. Furthermore, the drop-outs had a lower PA level at baseline and this could indicate that the intervention was more suitable for those who initially engaged in a minimum of PA. Because we did not ask when the participants performed their last exercise session, we do not know the extent to which the effects on insulin were the result of a long-term adaptation to increased PA or an acute response to one session. On the other hand, we have no information to suggest group differences in time since last session of PA.

Because ethnic minority populations show patterns and determinants of PA that are different from those of the majority population, duplicating successful interventions that target other groups (i.e. Caucasians) may therefore not be adequate to ensure success. Using a participatory approach, where representatives from the targeted community participate in the planning and development of the intervention seems to be essential to ensure success [18]. To the best of our knowledge, this study is the first randomised study to target PA behaviour in an immigrant population of SA origin. Since we included a combination of individual and group counselling sessions and organised exercise, we do not know the degree of contribution of the various components. The study was based on an individually adapted health behaviour change programme that used a SCT framework and was tailored to each individual’s readiness for change, specific interests and preferences. This programme had a goal of enabling the participants to incorporate more PA into their daily routine. A systematic review that included studies with approaches similar to those used in the present study showed a median net increase of 35 % in PA level in the 18 studies investigated [28]. None of these studies used accelerometers as an objective measure of PA, thereby making it difficult to compare the results. Furthermore, none of these studies were conducted with Asian immigrants. Generally, very few studies on the effectiveness of PA promotion interventions have targeted or included substantial numbers of ethnic minorities in order to draw any conclusions about their effect on these groups. The few studies that have focused on immigrant groups/ethnic minorities seem to have had little or no success in increasing the PA level of the participants [20]. Adherence to a physically active lifestyle over the long term is essential to derive sustainable health effects. However, developing interventions where the PA behaviour is maintained over the long term has proven challenging. In the current paper we report on the short-term effect of the intervention on the PA level but in another paper, soon to be published in International journal of behavioural nutrition and physical activity, we presents results from a 6 months follow-up and show that the PA level was maintained.

In insulin resistance, the ability of insulin to stimulate glucose disposal is impaired and compensated for by an increase in insulin secretion from the beta cells of the pancreas. In response to prolonged insulin resistance and the resultant hyperinsulinaemia, progressive beta-cell failure can occur, which in turn can lead to hyperglycaemia and finally diabetes. South Asians are known to be more insulin resistant than other ethnic groups [2] and it is hypothesised that the high prevalence of diabetes in SA communities is due to their increased susceptibility to developing insulin resistance [6]. The reason for this excessive insulin resistance among SA people is not known. However, lack of PA, an unhealthy diet followed by obesity, and specific genotypes have been hypothesised as plausible factors [2]. Individuals with an impaired glucose tolerance may initially have high insulin levels, and be at an increased risk for T2D. However, in the course of time the insulin levels will gradually decrease in T2D participants because of a production failure [29], meaning that our healthy participants are indeed a high-risk population for T2D, since 96 % of the participants in the intervention group had above-normal insulin-2 h values at baseline.

In the present study the intervention group reduced their insulin-2 h levels, which most likely means an important reduced constraint on the insulin-secreting cells of the pancreas. However, there were no changes in the HOMA-IR score. As seen in other studies [30, 31], despite this appreciable reduction in postprandial insulin response, blood glucose concentrations were slightly reduced. Long-term exercise studies have been shown to be relatively effective in reducing the postprandial insulin load [32–34]. Furthermore, even very light post-meal physical activity may blunt the postprandial blood glucose increase [35]. Additionally, a single session of strength exercise reduced the blood glucose response to carbohydrate intake 24 h later [36]. These studies suggest that there may be appreciable short-term effects of PA upon blood glucose and insulin, possibly to be reflected in the long-term effects observed in the present work. However, further studies are required to elucidate whether there is a relationship between acute and long-term blood glucose and insulin responses to PA.

The observed high baseline insulin values give a high intervention potential that may partly explain the positive results. Regression analyses have suggested that this intervention effect on insulin can be explained in part by an increased level of PA and a reduced inactive time, rather than a reduction in waist circumference. Balkau et al. [37] also found an association between PA and insulin, and the relationship between total PA and insulin sensitivity remained after controlling for BMI. The strength of this latter study was the use of an accelerometer in combination with the hyperinsulinaemic-euglycaemic clamp technique for measuring insulin sensitivity. Additionally, Chandalia et al. [6] demonstrated that Asian Indian men are less insulin sensitive than Caucasians, regardless of their level of total body fat. These results suggest that PA level and not fat mass may be the most important factor in determining insulin sensitivity. Long-term engagement in PA may influence insulin sensitivity in many ways, i.e. by increasing the sensitivity to insulin at the receptor level, and/or by promoting transduction of the insulin signal to various intracellular processes. Our study does not, however, elucidate the mechanisms behind the observed changes in insulin-2 h values.

## Conclusions

Using the present SCT-based PA programme, it is possible to achieve increases in PA and a reduction in serum insulin and thereby presumably reduce the risk of T2D in Pakistani immigrant men.
